# FROM PILOT TO PRODUCT: EVOLUTION OF A REMINISCENCE THERAPY INTERVENTION FOR PERSONS LIVING WITH DEMENTIA

**DOI:** 10.1093/geroni/igac059.2159

**Published:** 2022-12-20

**Authors:** Silvia Orsulic-Jeras, Farida Ejaz, Miriam Rose, Sara Powers, Ashlee Cordell, Christa Wilk, Lisbeth Sanders

**Affiliations:** Benjamin Rose Institute on Aging, Cleveland, Ohio, United States; Benjamin Rose Institute on Aging, Cleveland, Ohio, United States; Benjamin Rose Institute on Aging, Cleveland, Ohio, United States; The Benjamin Rose Institute on Aging, Cleveland, Ohio, United States; Benjamin Rose Institute on Aging, Cleveland, Ohio, United States; Benjamin Rose Institute on Aging, Cleveland, Ohio, United States; LifeBio Inc., Marysville, Ohio, United States

## Abstract

Reminiscence therapy (RT) and life story work are effective techniques for eliciting engagement in persons living with dementia (PLWD) and helping inform direct care staff about residents’ lives and preferences to facilitate quality person-centered care. However, there are challenges in bringing such programs to scale in a feasible and cost-effective way. Two studies were conducted to test development and evolution of an RT program, LifeBio Memory. A pilot study of the original LifeBio protocol was conducted with 238 residents in 16 Ohio nursing homes. Most residents (88%) indicated at posttest that they enjoyed telling their life stories, and 87% would recommend the program to other residents. However, manually gathering and processing life story data led to challenges with feasibility. Subsequently, novel software was developed to streamline the process using machine learning and artificial intelligence via a tablet application. Acceptability and feasibility were examined in two rounds, 9 months apart, of seven focus groups (n=35) conducted with PLWD, family care partners, and residential care staff. Audiotapes of the groups were transcribed; thematic data analysis was used to generate a list of recommended changes and showed high levels of acceptability and feasibility. Based on these results, it was determined that LifeBio Memory was preferred for obtaining life story information over the previous manual method (e.g., handwriting life story notes). Potential barriers to implementing the new platform in residential care settings were also identified. Implications include the importance of maintaining person-centered care practices when creating technological solutions to address PLWD needs.

